# Chest X-Ray–Based Telemedicine Platform for Pediatric Tuberculosis Diagnosis in Low-Resource Settings: Development and Validation Study

**DOI:** 10.2196/51743

**Published:** 2024-07-01

**Authors:** Juan J Gómez-Valverde, Ramón Sánchez-Jacob, José Luis Ribó, H Simon Schaaf, Lara García Delgado, Alicia Hernanz-Lobo, Daniel Capellán-Martín, Ángel Lancharro, Orvalho Augusto, Alberto L García-Basteiro, Begoña Santiago-García, Elisa López-Varela, María J Ledesma-Carbayo

**Affiliations:** 1 Biomedical Image Technologies ETSI Telecomunicación Universidad Politécnica de Madrid Madrid Spain; 2 Centro de Investigación Biomédica en Red de Bioingeniería, Biomateriales y Nanomedicina (CIBER-BBN) Madrid Spain; 3 Department of Radiology Children’s National Hospital & George Washington University School of Medicine Washington, DC United States; 4 Hospital Universitari General de Catalunya Barcelona Spain; 5 Desmond Tutu TB Centre, Department of Paediatrics and Child Health Faculty of Medicine and Health Sciences Stellenbosch University Cape Town South Africa; 6 Pediatric Infectious Diseases Department Gregorio Marañón University Hospital Madrid Spain; 7 Gregorio Marañón Research Health Institute (IiSGM) Madrid Spain; 8 Centro de Investigación Biomédica en Red de Enfermedades Infecciosas (CIBERINFEC) Instituto de Salud Carlos III Madrid Spain; 9 RITIP Translational Research Network in Pediatric Infectious Diseases Madrid Spain; 10 Radiología Pediátrica Hospital Materno Infantil Gregorio Marañón Madrid Spain; 11 Radiología Pediátrica, HM Hospitales Madrid Spain; 12 Centro de Investigacão em Saúde de Manhiça (CISM) Maputo Mozambique; 13 Department of Global Health University of Washington Seattle, WA United States; 14 ISGlobal, Hospital Clínic Universitat de Barcelona Barcelona Spain

**Keywords:** telemedicine, telehealth, pediatric tuberculosis, tuberculosis, screening, chest radiograph, usability, low-resource settings

## Abstract

**Background:**

Tuberculosis (TB) remains a major cause of morbidity and death worldwide, with a significant impact on children, especially those under the age of 5 years. The complex diagnosis of pediatric TB, compounded by limited access to more accurate diagnostic tests, underscores the need for improved tools to enhance diagnosis and care in resource-limited settings.

**Objective:**

This study aims to present a telemedicine web platform, BITScreen PTB (Biomedical Image Technologies Screen for Pediatric Tuberculosis), aimed at improving the evaluation of pulmonary TB in children based on digital chest x-ray (CXR) imaging and clinical information in resource-limited settings.

**Methods:**

The platform was evaluated by 3 independent expert readers through a retrospective assessment of a data set with 218 imaging examinations of children under 3 years of age, selected from a previous study performed in Mozambique. The key aspects assessed were the usability through a standardized questionnaire, the time needed to complete the assessment through the platform, the performance of the readers to identify TB cases based on the CXR, the association between the TB features identified in the CXRs and the initial diagnostic classification, and the interreader agreement of the global assessment and the radiological findings.

**Results:**

The platform’s usability and user satisfaction were evaluated using a questionnaire, which received an average rating of 4.4 (SD 0.59) out of 5. The average examination completion time ranged from 35 to 110 seconds. In addition, the study on CXR showed low sensitivity (16.3%-28.2%) but high specificity (91.1%-98.2%) in the assessment of the consensus case definition of pediatric TB using the platform. The CXR finding having a stronger association with the initial diagnostic classification was air space opacification (χ21>20.38, *P*<.001). The study found varying levels of interreader agreement, with moderate/substantial agreement for air space opacification (κ=0.54-0.67) and pleural effusion (κ=0.43-0.72).

**Conclusions:**

Our findings support the promising role of telemedicine platforms such as BITScreen PTB in enhancing pediatric TB diagnosis access, particularly in resource-limited settings. Additionally, these platforms could facilitate the multireader and systematic assessment of CXR in pediatric TB clinical studies.

## Introduction

Tuberculosis (TB) is a communicable disease caused by *Mycobacterium tuberculosis*. According to the World Health Organization (WHO), TB remains one of the leading causes of death globally from a single infectious agent, with over 1.6 million TB-related deaths reported in 2021 [1]. Alarmingly, most children who succumb to TB are never diagnosed or treated [2]. The risk of death is notably high (44%) among children under 5 years with untreated TB, while less than 1% of children receiving recommended treatment die [3].

The diagnosis of TB in children is complex, especially in infants and young children, where the risk of rapid disease progression and mortality is higher than in any other age group [4,5]. The paucibacillary nature of TB in this age group and the absence of highly sensitive point-of-care diagnostic tests to microbiologically confirm pediatric TB make diagnosis challenging [4]. Chest x-ray (CXR) remains a valuable diagnostic tool for TB in children, especially when laboratory testing is unavailable, infeasible, or yields negative results. Most children with pulmonary TB exhibit radiographic changes indicative of TB. For children under 5 years, anteroposterior (AP) and lateral (LAT) views are recommended, while posteroanterior (PA) CXRs are preferred for older children and adolescents [6]. The LAT radiograph is particularly useful in children under 5 years for the optimal evaluation of hilar or mediastinal lymphadenopathy [7]. CXR findings in children with pulmonary TB may lack specificity [8], and CXR alone is insufficient to determine the appropriate treatment for the child. Instead, CXR can support the clinical diagnosis of pulmonary TB when TB is presumed and microbiological testing is negative.

Screening tests using symptoms or CXR may be useful in children who are TB contacts or living with HIV [2]. According to Vonasek et al [2], any abnormality identified on CXR appears to be the most accurate screening test for pulmonary TB in children, although this accuracy can be influenced by the quality of the CXR and interreader variability. In a recent study [9] involving a cohort of HIV-negative children, the majority of whom (92%) were under 5 years old, a treatment-decision algorithm was proposed for low-resource countries. In these settings, CXRs are reserved to confirm diagnoses in patients lacking sufficient clinical evidence to initiate treatment. The WHO guidelines underscore the necessity for further research concerning integrated treatment-decision algorithms [6]. This highlights the crucial importance of promoting research aimed at improving and validating these tools within the pediatric context, thus facilitating informed recommendations in this area [9,10].

Assessing disease severity in children is essential for determining their eligibility for the recommended 4-month treatment regimen for nonsevere TB in children and adolescents aged 3 months to 16 years. CXRs serve as a valuable tool for this purpose. Furthermore, recent WHO guidelines [6] emphasize that CXRs can assist in evaluating treatment response and identifying alternative diagnoses in children who do not respond to TB treatment.

The limitations in accessibility and sensitivity of available diagnostic tests for childhood TB are probable reasons for the gap between the estimated 1.17 million annual incident child TB cases, of which less than half are diagnosed or reported to the WHO [6]. This gap is even more pronounced for children under 5 years old. Additionally, the COVID-19 pandemic has decreased access to TB diagnosis and treatment, particularly affecting children and young adolescents, resulting in a significant decrease in notifications for younger age groups. To address these challenges, the End TB Strategy outlined by the WHO emphasizes the importance of leveraging enhanced digital health tools for more efficient delivery, monitoring, and evaluation of TB patient diagnosis, treatment, and care [11,12]. Telemedicine tools could play a crucial role in enhancing accessibility for diagnosis and treatment. Previous studies have shown that telemedicine can be beneficial in optimizing the care of multidrug-resistant TB in resource-limited settings [13]. Moreover, providing specialist expertise directly through telemedicine tools in low-resource settings has not only improved patient management but also provided additional educational value to local physicians, thereby benefiting other patients as well [14].

In this paper, we introduce a novel telemedicine web platform called BITScreen PTB (Biomedical Image Technologies Screen for Pediatric Tuberculosis), designed for the assessment of pediatric TB using digital CXR images and clinical information. The platform aims to facilitate remote interpretation, streamlining, and standardizing the clinical evaluation of pediatric TB cases, particularly in resource-limited settings where access to expert readers may be limited. The platform underwent functional evaluation in a pilot study conducted by 3 independent expert readers (RSJ, JLR, and HSS). This evaluation involved a retrospective assessment of a data set comprising 218 examinations of children under 3 years of age, selected from a previous study conducted in Mozambique [5,10]. Furthermore, based on the results of the evaluations conducted through the platform in the pilot study, we present new insights into its performance, the agreement among evaluators, and the challenges associated with the assessment of pediatric TB using CXR images, considering various radiological findings.

## Methods

### BITScreen Platform

BITScreen is a store-and-forward telemedicine platform built using a Model-View-Controller (MVC) design pattern, implemented on open-source frameworks and tools by JJGV. The MVC design pattern offers a modular and scalable structure for organizing and building software applications, facilitating efficient development, maintenance, and expansion of the platform. In an MVC application, the “View” is responsible for presenting information to the end user, while the “Controller” manages the user’s interaction using the data stored and organized in the “Model.” The primary functional requirement of the platform is to facilitate asynchronous medical evaluation of pediatric TB studies. This involves assessing clinical data and CXR images, optionally including corresponding clinical symptoms. The global requirements identified in the design of the system are listed in Textbox 1.

**Global requirements identified in the system design.**

- Multistudy

The capacity to perform multiple clinical projects simultaneously.

- Multicenter

The system must allow the participation of multiple medical centers and admit many-to-many relationships between medical centers and projects/studies.

- Multidevice

Web-based access to the views of the platform, which allows its use in different devices through an internet browser.

- Security

The platform must warrant security in terms of authentication, confidentiality, and integrity in compliance with European regulations.

- Cloud Storage

The system must enable the secure storage of images, tests, and reports associated with the project in a remote environment.

**Figure 1:**
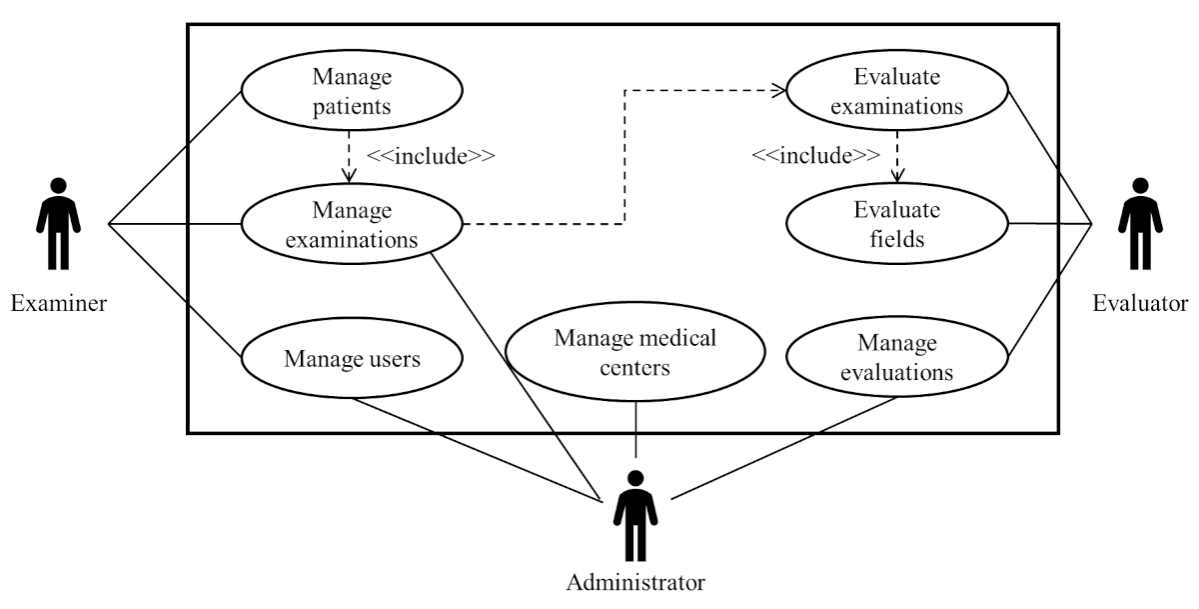
Use case diagram of the BITScreen PTB (Biomedical Image Technologies Screen for Pediatric Tuberculosis) platform with the 3 roles considered (examiner, evaluator, and administrator) and the operations associated with them. All the “Manage” operations included the suboperations new, edit, and delete.

Figure 1 illustrates the unified modeling language use case diagram, which delineates the interaction between users and the system. The user roles include the examiner, responsible for patient management and creating new examinations; the evaluator, tasked with assessing studies by identifying potential TB-related findings in CXR images; and the administrator user, responsible for managing user and medical center access. Additionally, the administrator defines examiners (individuals who examine patients) and evaluators (individuals who assess CXRs) and monitors the progress of evaluations.

**Figure 2:**
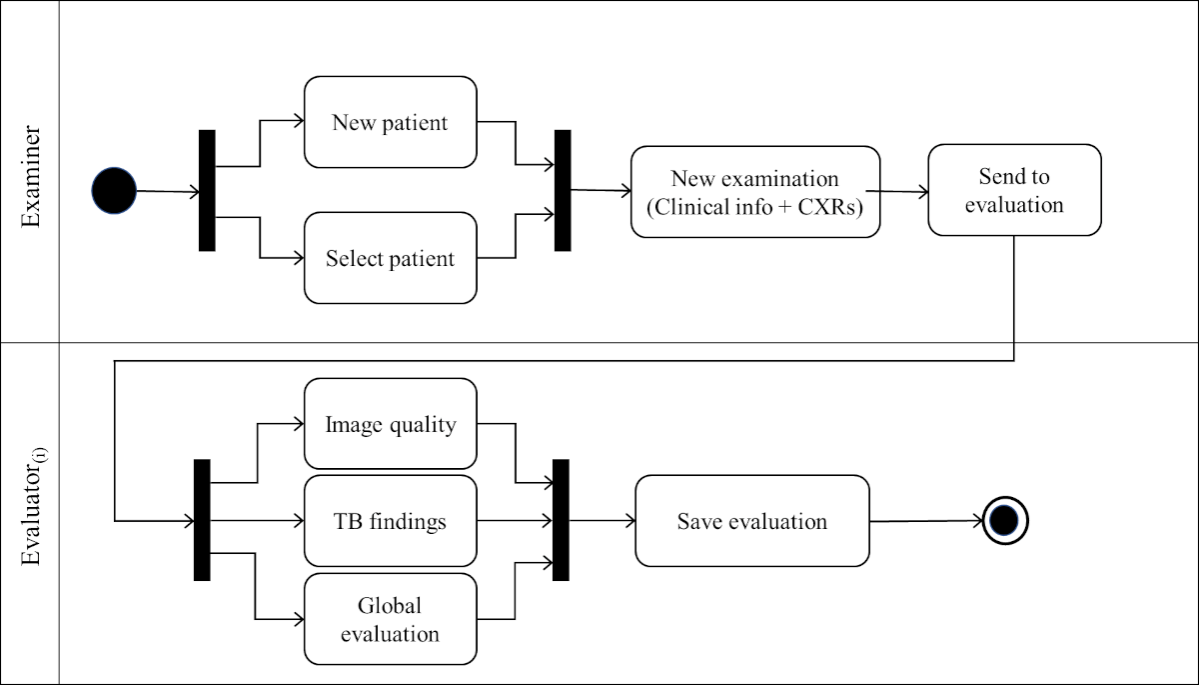
Activity diagram of the process for creating and evaluating a new examination including the clinical information about the patient and the chest x-ray (CXR) images (anteroposterior or posteroanterior and lateral views). TB: tuberculosis.

Figure 2 displays the activity diagrams designed to illustrate the process of uploading a new examination to the platform by an examiner, incorporating clinical information and CXR images, as well as the subsequent transmission of the corresponding examination to be evaluated by an evaluator user. The input fields included by the examiner to create a new examination were month and year of birth, date of the examination, cough, fever, malnutrition, HIV status, BCG (Bacillus Calmette-Guérin) vaccine scar, tuberculin skin test, TB diagnosis, TB contact, TB treatment, treatment starting date, and the CXR images (AP or PA and LAT views). In our pilot study, only the CXR images were presented to the evaluators. The patient’s age was determined based on the month and year of birth in relation to the acquisition date of the CXR. The examiner is required to upload at least one AP or PA view CXR image, with the LAT view being optional if available. In this pilot study, only evaluators were granted access to the CXR images. In the evaluation process, the platform was designed to include the assessment of the image quality of the CXR images; the identification of pulmonary TB radiological findings in various regions of the lungs, considering different types of findings; and a global evaluation of the CXR examination. In Figure 2, only 1 evaluation is depicted, but the platform allows for multiple evaluations (in our platform validation, we included 3 evaluations for each examination). If more than 1 evaluation is configured, the evaluation process of the examination will not conclude until all evaluators have completed their assessments within the platform.

**Figure 3:**
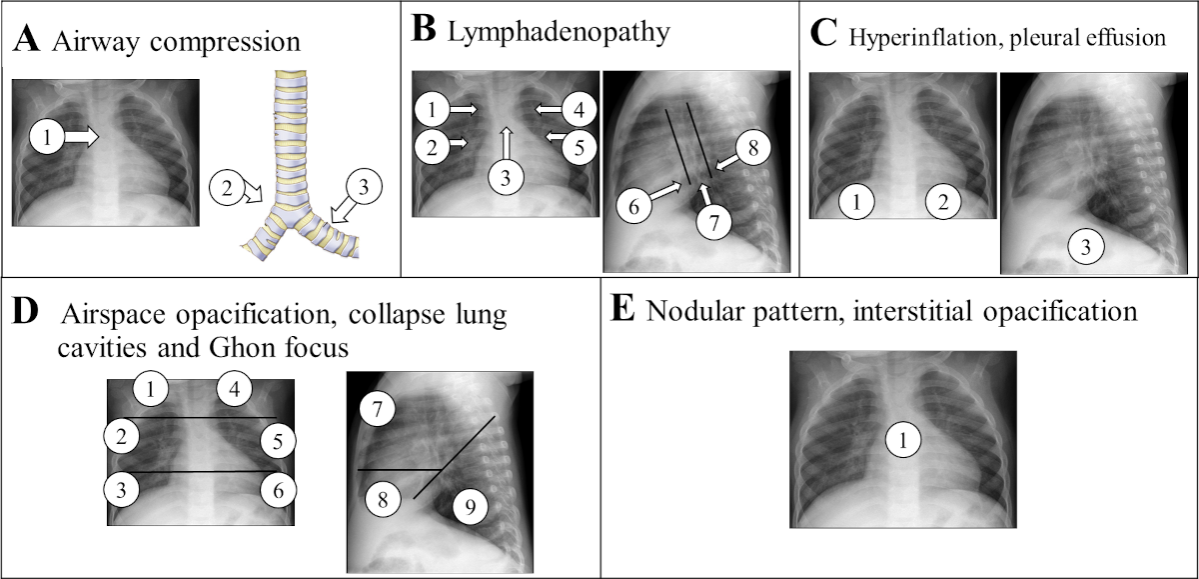
Evaluation templates with the location of the specific findings that should be assessed by the evaluators with “yes” or “no” for each of the 10 sections. (A) Locations for the evaluation of possible airway compression or tracheal displacement. (B) Locations for the assessment of soft tissue density suggestive of lymphadenopathy. (C) Locations for the assessment of hyperinflation and pleural effusion. (D) Locations for the evaluation of air space opacification, collapsed lung, cavities, and calcified parenchyma. (E) Location for the assessment of nodular pattern, either miliary or larger widespread and bilateral nodules, and interstitial opacification. Based on [15,16] and chest x-ray review tool developed by Andronikou and the South African Tuberculosis Vaccine Initiative and used in Graham et al [16].

The evaluation of CXR images is pivotal for identifying presumed patients with TB and constitutes a primary focus of the platform’s design. To ensure a comprehensive and rigorous assessment of the CXR images, evaluators are required to indicate “yes” or “no” to assess the presence or absence of various radiological TB findings across different thoracic locations. For this purpose, we divided the assessment into 10 sections corresponding to different types of findings, resulting in a total assessment of 55 independent observations, with 36 from the AP/PA view and 19 from the LAT view. The 10 sections of pediatric CXR TB findings corresponded to airway compression or tracheal displacement, soft tissue density suggestive of lymphadenopathy, hyperinflation, pleural effusion, air space opacification, collapsed lobe or lung, cavities, calcified parenchymal lesions, nodular pattern, and interstitial opacification. Figure 3 depicts the templates provided to the evaluators, highlighting the specific locations of the features to be assessed. These locations and types of findings were determined based on previous recommendations in the literature, including the “Diagnostic CXR Atlas for Tuberculosis in Children” [15] and the CXR review tool developed by Andronikou and the South African Tuberculosis Vaccine Initiative (SATVI) and used in Graham et al [16].

For the back-end implementation of the platform, we used PHP’s Laravel framework (version 6.2; PHP Group). Laravel offers a range of built-in tools and features, which were leveraged in the project, including routing, authentication, authorization, database connection management, and the Blade templating engine. For data storage, we opted for the MariaDB database (version 10.1.38; MariaDB plc/MariaDB Foundation), a fork of the MySQL database management system. We chose MariaDB because of its efficiency, customization options, portability, reliability, open-source nature, cost-effectiveness, and widespread adoption by a large and active community. The front end of the platform was built using the Bootstrap framework (version 4.3.1; Bootstrap Core Team), which offers a plethora of predesigned components that can be seamlessly integrated into a website. Indeed, Bootstrap’s responsive design ensures that the application can be easily accessed and used across a wide range of devices and screen sizes. As for the server configuration, it operates on Debian 4.9 (Debian Project) and is equipped with 2 virtual central processing unit cores (Intel Xeon), 4 GB of RAM, and 100 GB of hard disk space. This setup provides a stable and efficient environment for hosting the platform and handling user interactions.

### Data Set Pilot Study

The data set used to evaluate the platform in our pilot study was sourced from a previous prospective descriptive study called ITACA [5]. This study focused on young children under 3 years of age presumed to have TB and was conducted at the Manhiça Health Research Center (CISM), situated in Southern Mozambique [5,10]. For our evaluation, we collected a total of 218 examinations. This included all microbiologically confirmed and “probable” cases, as well as a random selection of 113 additional cases from the unlikely TB cases subset. The cases were confirmed using Ziehl-Neelsen staining, rapid tests, and Xpert MTB/RIF, with identification through mycobacterial molecular identification (HAIN GenoType Mycobacterium CM/AS; Hain Lifescience) [5]. Table 1 presents their demographic data. To enhance comparability between studies and encourage the standardization of diagnostic procedures, we adhered to the case definition classification for research reporting based on diagnostic evaluation studies of intrathoracic TB in children proposed by Graham et al [17]. In this update from the previous case definitions presented in 2012 and 2013 [16,18], the authors established 3 case definitions: confirmed TB, unconfirmed TB, and unlikely TB. The collected cases were retrospectively classified [5] according to these definitions, using the information gathered from the previous study [10]. Table 2 displays the TB diagnosis categories identified alongside the corresponding clinical data for each case. The symptom definitions considered were as follows [5]: cough for 14 days or more not responding to a course of antibiotics; fever greater than 38°C for 14 days or more; malnutrition defined as under 60% weight for height, failure to gain weight for more than 2 months, or any loss of weight not responsive to nutritional intervention; and TB contact in the last 12 months.

**Table 1:** Patient demographic characteristics of the data set of the pilot study.

Demographic characteristics		Male (n=122), n (%)	Female (n=96), n (%)	Total (N=218), n (%)
**Age range**				
	<12 months	18 (14.8)	19 (19.8)	37 (17.0)
	12-23 months	54 (44.3)	43 (44.8)	97 (44.5)
	24-35 months	50 (41.0)	34 (35.4)	84 (38.5)

**Table 2:** Diagnostic categories and corresponding clinical characteristics considering the definitions from López-Varela et al [5] and Graham et al [17].

TB^a^ category		Confirmed (n=10), n (%)	Unconfirmed TB (n=95), n (%)	Unlikely TB (n=113), n (%)
**Sex**				
	Female	6 (60.0)	44 (46.3)	46 (40.7)
**Age range (months)**				
	<12	3 (30.0)	21 (22.1)	13 (11.5)
	13-23	3 (30.0)	42 (44.2)	52 (46.0)
	24-35	4 (40.0)	32 (33.7)	48 (42.5)
**Cough**				
	Yes	5 (50.0)	17 (17.9)	14 (12.4)
**Fever**				
	Yes	4 (40.0)	6 (6.3)	5 (4.4)
**Malnutrition**				
	Yes	5 (50.0)	81 (85.3)	104 (92.0)
**HIV status**				
	Positive	2 (20.0)	35 (36.8)	4 (3.5)
**BCG^b^ scar**				
	Yes	10 (100.0)	94 (98.9)	113 (100.0)
**TB contact**				
	Yes	2 (20.0)	11 (11.6)	4 (3.5)

^a^TB: tuberculosis.

^b^BCG: Bacillus Calmette-Guérin.

### Ethics Approval

The ITACA study protocol received approval from both the Mozambican National Bioethics Committee (15/CNBS) and the Hospital Clinic of Barcelona Ethics Review Committee (HCB/2009/4682). Written informed consent was obtained from the parents/legal guardians of all study participants. Additionally, the substudy focusing on the digital processing of the CXR images was approved by the Mozambican National Bioethics Committee.

### Evaluation Protocol

The 218 baseline examinations, conducted at the time of evaluation for presumptive TB, were uploaded by the administrator user using the platform’s automatic importing feature. This was done via a CSV file containing the input fields outlined in Table 2, along with the location of the CXR files featuring the AP view (in all participants) and the LAT view (in 207 participants). The platform automatically assigned all cases to 3 pediatric CXR expert readers, each possessing extensive experience in assessing TB imaging in endemic settings of low-income, resource-limited countries [5,19]. These 3 evaluators conducted a blind evaluation of the 218 examinations using the platform, relying solely on the CXR views and reference templates (Figure 3), without any additional information. The evaluation encompassed the following components: (1) assessment of CXR image quality, categorized as “acceptable,” “poor but readable,” or “not acceptable not readable”; (2) evaluation of 55 observations across 10 sections, with responses marked as “yes” or “no”; and (3) a final global evaluation of the case, categorized as “suggestive of TB,” “not suggestive of TB,” or “not evaluable.”

### Performance Metrics

To evaluate the performance of the evaluations, we used the metrics sensitivity, specificity, positive predictive value (PPV), *F*_1_-score, and accuracy. We defined sensitivity or recall as the number of true-positive cases with x-ray findings suggestive of TB divided by the sum of true positives and false negatives. We defined specificity as the number of true negatives divided by the sum of true negatives and false positives. The PPV is the proportion of true-positive predictions out of all positive predictions (true positives + false positives). It measures how many of the positive predictions are actually correct. The *F*_1_-score serves as a measure of a model’s accuracy by blending both the PPV and recall. It is commonly used to assess the effectiveness of a classification algorithm. Ranging between 0 and 1, an *F*_1_-score of 1 indicates flawless PPV and recall, while a score of 0 signifies the poorest performance achievable. Accuracy, by contrast, is calculated as the sum of true positives and true negatives divided by the sum of true positives, true negatives, false positives, and false negatives. A true-positive case is identified when an evaluator marks a case as “suggestive of TB” in the global evaluation, and the examination is classified as either “confirmed” or “unconfirmed TB.” Conversely, a true-negative case occurs when the evaluator designates “not suggestive of TB,” and the examination is labeled as “unlikely TB.” An examination is considered a false negative if the evaluator indicates “not suggestive of TB,” yet the case is classified as “confirmed” or “unconfirmed TB.” A case is classified as false positive if an evaluator marks it as “suggestive of TB,” while the examination is categorized as “unlikely TB.” Furthermore, we examined the relationship between TB features identified in the CXRs and the global evaluation (“suggestive of TB” and “not suggestive of TB”) concerning the initial diagnostic classification, combining “confirmed” and “unconfirmed TB.” Statistical significance was determined by a chi-square *P* value <.05. Finally, we used Cohen kappa to assess the interreader agreement across all evaluations conducted by the evaluators, including CXR image quality, TB feature assessments, and TB global evaluations. Kappa scores were categorized as follows: ≤0 for no agreement, 0.01-0.2 for slight agreement, 0.21-0.4 for fair agreement, 0.41-0.6 for moderate agreement, 0.61-0.8 for substantial agreement, and 0.81-1.00 for almost perfect agreement.

### Platform Usability Evaluation

We developed a comprehensive questionnaire comprising 5 sections and 15 items to thoroughly assess the usability of the platform. This questionnaire was adapted from the Telehealth Usability Questionnaire (TUQ) proposed by Parmanto et al [20], a well-established tool for evaluating telemedicine services [21]. Our questionnaire addresses various crucial usability aspects, encompassing usefulness (3 items), ease of use and learnability (2 items), interface quality (4 items), reliability (2 items), and global satisfaction (2 items). A detailed breakdown of the questionnaire components and associated items is presented in Table 3. Additionally, we conducted an analysis to ascertain the duration of the evaluation process for each examination. We precisely measured the duration from the initiation of a new examination request to the submission of the evaluator’s final evaluation into the system. By computing the time difference between these 2 events, we obtained a precise and dependable estimate of the time taken by the expert to conduct a comprehensive evaluation of an examination.

## Results

**Figure 4:**
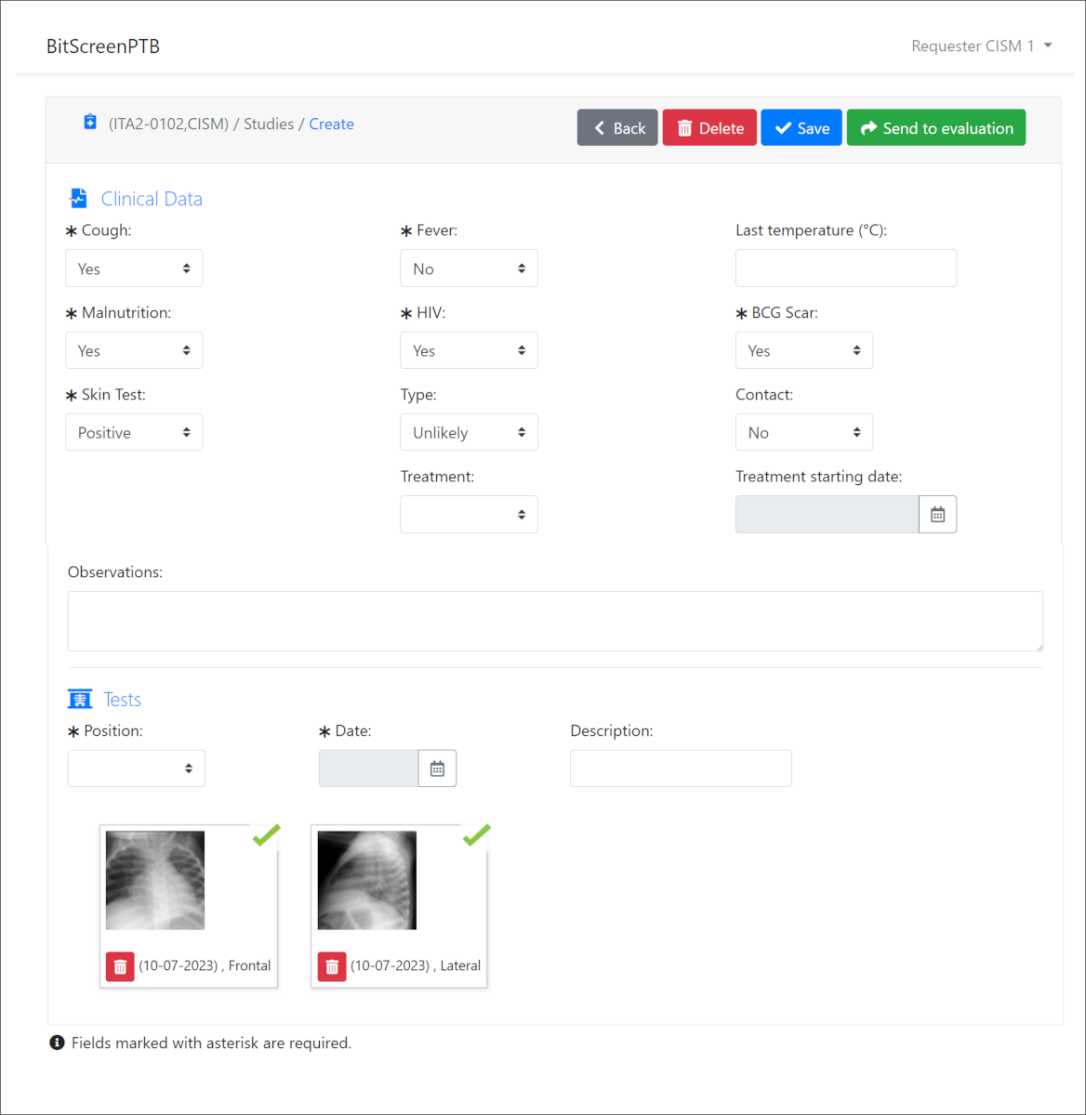
Example of the BITScreen PTB (Biomedical Image Technologies Screen for Pediatric Tuberculosis) examiner user view of a new examination with the 2 different areas: Clinical data and Images.

**Figure 5:**
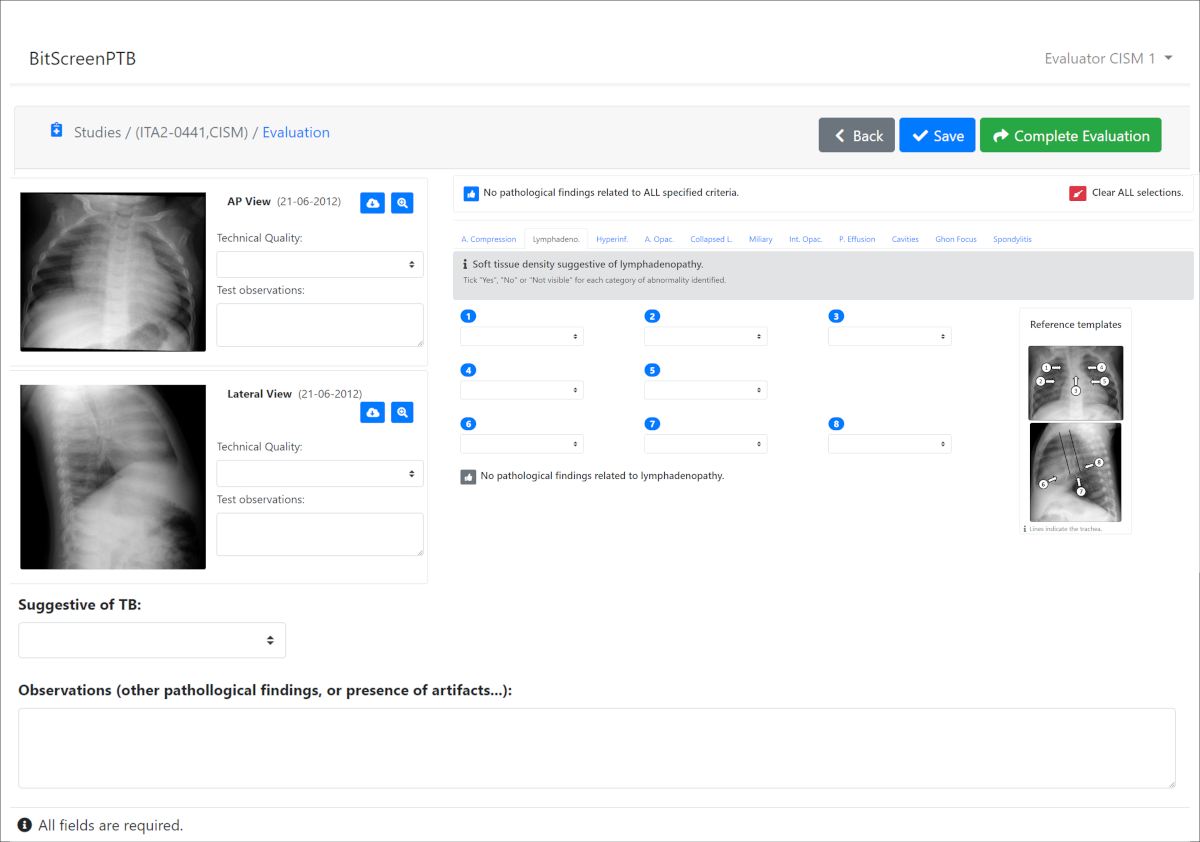
Example of the BITScreen PTB (Biomedical Image Technologies Screen for Pediatric Tuberculosis) evaluator user view with 3 different areas: quality image assessment, identification of the presence of findings in the different locations presented in the templates, and a global evaluation of the case.

The 2 primary views of the new BITScreen platform are illustrated in Figures 4 and 5: the input form utilized by examiner users and the evaluation form used by evaluator users, respectively. In the top section of the input form (Figure 4), examiners input details such as cough, fever, last temperature, malnutrition, HIV status, BCG scar presence, tuberculin skin test result, TB category, contact with a TB source patient, treatment status, treatment starting date, and any observations. In the bottom section, examiners have the option to upload CXR images for evaluation by the evaluators.

In the case of the evaluation form (Figure 5), the view used by the evaluators displays the CXR images on the left side of the screen, allowing them to download or zoom in on each image for detailed examination. Evaluators are tasked with assessing the quality of each CXR image. On the right side of the screen, the 10 sections described previously are presented as separate tabs. Within these tabs, evaluators are required to assess all 55 different observations. The templates depicted in Figure 3 remain consistently visible in the view to aid evaluators in their tasks. Readers are provided with the option to mark all locations without pathological findings as “no” for all criteria at once or for all locations of a specific criterion, streamlining and expediting the evaluation process. At the bottom of the view, the global evaluation field for the examination is displayed. All fields are mandatory, except in cases where the CXR images are deemed not evaluable.

The results of the usability questionnaire administered on the telemedicine platform are outlined in Table 3. The overall score for all questions averaged 4.4 (SD 0.59) out of 5. Users rated the platform positively in terms of usefulness, with an average rating of 4.42 out of 5; ease of use and learnability, receiving an average rating of 4.47 out of 5; and interface quality, which garnered positive feedback with an average rating of 4.13 out of 5. The platform was also perceived as reliable, with an average rating of 4.26 out of 5 and a high level of variability (SD 0.82). Additionally, all 3 evaluators expressed a high level of satisfaction with the platform, giving it an average rating of 5.0 out of 5.

Some specific questions received lower ratings, particularly item 4 in the interface quality dimension (The system is able to do everything I would want it to be able to do) and item 1 in the reliability dimension (Whenever I made a mistake using the system, I could recover easily and quickly). Conversely, items with higher feedback included item 1 in the ease of use and learnability section (It was simple to use this system), as well as questions related to global satisfaction and future use, where “I would use the platform again” and “Overall, I am satisfied with the platform” received maximum feedback from all evaluators.

**Table 3:** Results of the Usability Questionnaire (1=strongly disagree to 5=strongly agree).

Section			Mean (SD)
**Usefulness**			4.42 (0.53)
	1. It facilitates the assessment of CXRs^a^ in pediatric TB^b^ studies	4.64 (0.58)	
	2. It saves me time assessing CXRs in pediatric TB studies	4.31 (0.58)	
	3. It includes all the items I need to evaluate pediatric TB studies	4.31 (0.58)	
**Ease of use and learnability**			4.47 (0.52)
	1. It was simple to use this system	5.00 (0.00)	
	2. It was easy to learn the system	4.31 (0.58)	
	3. The templates with the location of the findings facilitate the assessment of the cases	4.31 (0.58)	
	4. I believe I could become productive quickly using this system	4.31 (0.58)	
**Interface quality**			4.13 (0.58)
	1. The way I interact with this system is pleasant	4.00 (0.00)	
	2. I like using the system	4.31 (0.58)	
	3. The system is simple and easy to understand	4.31 (0.58)	
	4. The system is able to do everything I would want it to be able to do	3.91 (1.00)	
**Reliability**			4.26 (0.82)
	1. Whenever I made a mistake using the system, I could recover easily and quickly	3.91 (1.00)	
	2. The system gave error messages that clearly told me how to fix the problems	4.64 (0.58)	
**Satisfaction and future use**			5.0 (0.00)
	1. I would use the platform again	5.0 (0.00)	
	2. Overall, I am satisfied with the platform	5.0 (0.00)	

^a^CXR: chest x-ray.

^b^TB: tuberculosis.

**Figure 6:**
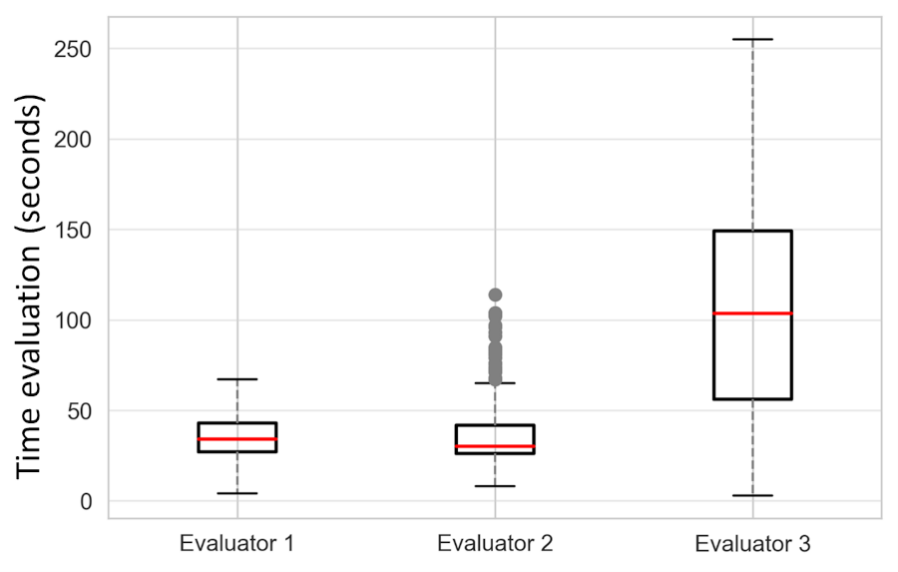
Evaluation time in seconds by the 3 evaluators of the 218 examinations.

Figure 6 presents the completion times of the evaluators. Evaluator 2 demonstrated the shortest completion time, averaging 35.3 (SD 13.2) seconds. Evaluator 1 followed with an average time of 37.8 (SD 19.2) seconds, while evaluator 3 recorded the longest completion time, averaging 110.3 (SD 63.2) seconds. Despite evaluator 3 taking more time, their superior performance and identification of more observations justify the additional time spent. A previous study [22] has indicated that radiologists typically spend an average of 2 minutes and 9 seconds (129 seconds) evaluating and reporting neonatal CXR images, a duration longer than what was observed in our study. However, it is crucial to highlight that our reviewers were tasked solely with marking specific findings’ locations, assessing image quality, and delivering a global assessment, without the need to compose a report or dictate findings. In any case, our findings suggest that the platform could serve as a valuable tool for swiftly evaluating cases and annotating findings in CXR images.

Next, we present the results from the assessment of 218 examinations in this pilot study. The evaluation of the CXR AP views revealed that 195/219 (89.0%), 167/193 (86.5%), and 150/219 (68.5%) images were rated as “acceptable” by evaluators 1, 2, and 3, respectively. Additionally, 23/219 (10.5%), 26/193 (13.5%), and 65/219 (29.7%) were rated as “poor but readable.” However, for the LAT views, the image quality was lower. Specifically, 160/209 (76.6%), 109/161 (67.7%), and 128/208 (61.5%) images were rated as “acceptable” by the 3 evaluators, while 42/209 (20.1%), 46/161 (28.6%), and 59/208 (28.4%) were rated as “poor but readable.” Additionally, 7/209 (3.3%), 6/161 (3.7%), and 21/208 (10.1%) LAT views were deemed “not acceptable, not readable.” Notably, only evaluator 3 rated all views of the CXRs as “not acceptable, not readable” in 2 examinations, and there was only 1 image that received this rating from all 3 evaluators. The number of images classified in each category by each expert is presented in Figure S1 in Multimedia Appendix 1, while Figure S2 in Multimedia Appendix 1 provides examples of images and their corresponding ratings.

**Figure 7:**
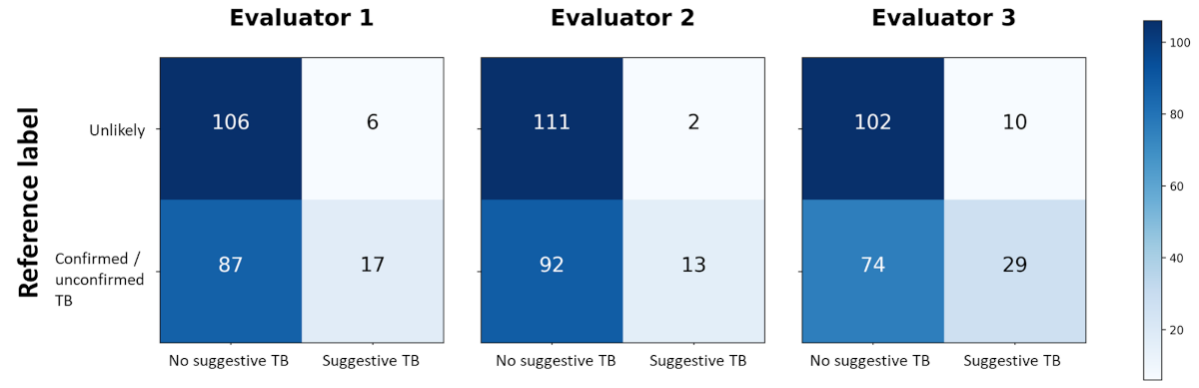
Confusion matrices of the 3 evaluators. TB: tuberculosis.

Table 4 displays the performance metrics of the global evaluation. Among the 3 evaluators, evaluator 3 exhibited the highest sensitivity (28.2%), *F*_1_-score (40.8%), and accuracy (60.9%). However, evaluator 3 had the lowest specificity (91.1%), indicating a potential tendency to classify more unlikely TB cases as suggestive of TB compared with the other evaluators. Evaluator 2 demonstrated the highest specificity (98.2%), suggesting proficiency in accurately identifying unlikely TB cases. However, the evaluator displayed the lowest scores for sensitivity (12.4%) and *F*_1_-score (21.7%), indicating challenges in correctly identifying both confirmed and unconfirmed TB cases. Evaluator 1’s scores were intermediate across all metrics, except for PPV, which exhibited the lowest score (73.9%). This suggests that while evaluator 1 did not excel in any specific metric, the performance was consistently average across all metrics. To further illustrate the results, Figure 7 showcases the confusion matrices with the corresponding counts of true negatives (top left), true positives (bottom right), false positives (top right), and false negatives (bottom left), while Table S1 in Multimedia Appendix 1 provides the evaluation for each TB diagnostic class.

**Table 4:** Performance metrics considering sensitivity, specificity, positive predictive value, *F*1-score, and accuracy (N=218)^a^.

Metrics	Evaluator 1	Evaluator 2	Evaluator 3
Sensitivity (95% CI)	16.3 (10.5-24.6)	12.4 (7.4-20.0)	28.2 (20.4-37.5)
Specificity (95% CI)	94.6 (88.8-97.5)	98.2 (93.8-99.5)	91.1 (84.3-95.1)
Positive predictive value (95% CI)	73.9 (50.3-63.4)	86.7 (62.1-96.3)	74.4 (58.9-85.4)
*F*_1_-score (95% CI)	26.8 (19.8-35.1)	21.7 (15.2-29.9)	40.8 (33.1-49.1)
Accuracy (95% CI)	56.9 (50.3-63.4)	56.9 (50.2-63.3)	60.9 (54.3-67.2)

^a^All values are in percentages.

**Figure 8:**
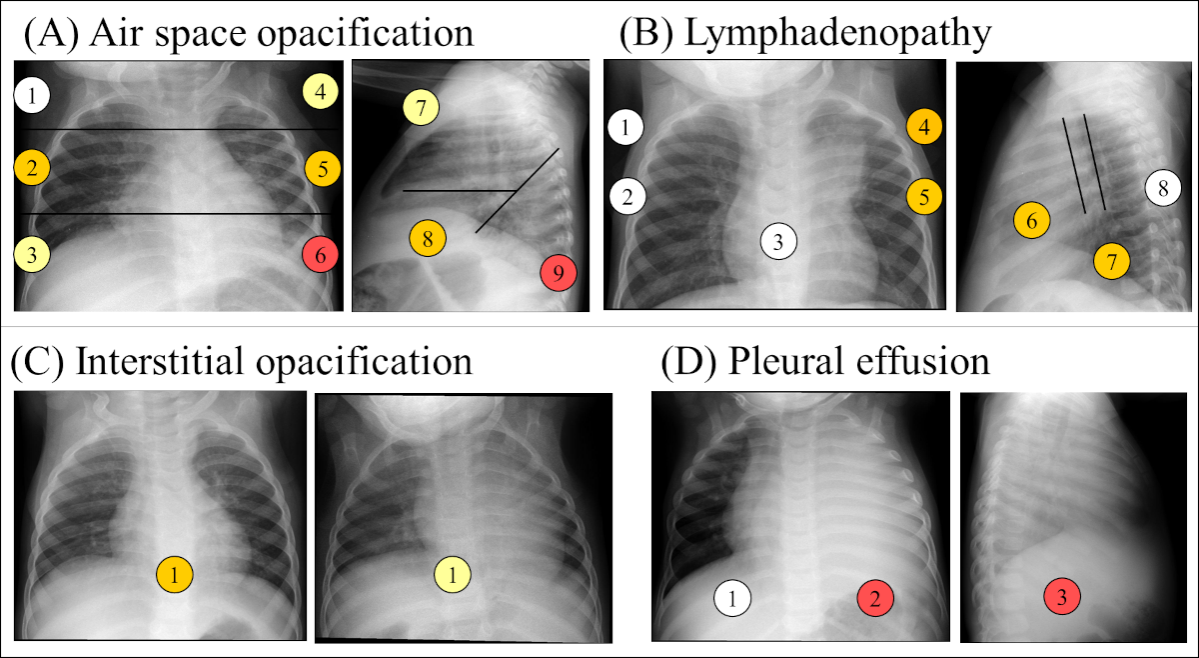
Example of evaluations of findings in different studies. The locations of the findings are defined in Figure 3. The color of the locations represents the number of evaluators that identified the presence of the finding in that location, being 0 evaluators for white, 1 evaluator for yellow, 2 evaluators for orange, and 3 evaluators for red. (A) Presence of air space opacification in the anteroposterior (AP) and lateral chest x-ray (CXR) views of an examination of a female patient of 11 months classified as unconfirmed tuberculosis (TB) and as suggestive of TB by 1 out of the 3 evaluators. (B) Presence of lymphadenopathy in the AP and lateral CXR views of an examination of a female patient of 11 months classified as confirmed TB and as suggestive of TB by the 3 evaluators. (C) Presence of interstitial opacification on AP CXR views of 2 studies, the one on the left is from a male patient of 1 year and 4 months of age. Both studies were classified as unconfirmed TB and not suggestive of TB. The AP view on the right corresponds to a patient of female of 11 months of age. The examination was classified as unlikely TB and 1 out of 3 evaluators assessed it as confirmed TB. (D) Presence of pleural effusion in the AP and lateral view of an examination of a male of 2 years and 2 months of age classified as confirmed TB and evaluated as suggestive of TB by the 3 evaluators.

Table 5 displays the number of observations recorded by each of the 3 evaluators in the 3 diagnostic categories, namely, confirmed TB, unconfirmed TB, and unlikely TB, across the 10 examination fields. The total number of observations recorded by the 3 evaluators was 64, 59, and 150, highlighting a substantial difference between evaluator 3 and the other 2 evaluators. This difference was particularly noticeable in the unconfirmed TB and unlikely TB categories. Air space opacification emerged as the category with the highest number of observations by all evaluators, notably in the unconfirmed TB category, where it ranged from 22 to 33, totaling 95 cases. Following closely, lymphadenopathy was the second most frequently observed area, with evaluator 3 recording this finding in 34 examinations across all categories, 22 of which were in the unconfirmed TB category. Additionally, a notable number of observations were recorded in the interstitial opacification field, with evaluator 3 being particularly active in marking this finding in 16 examinations. By contrast, the areas of cavities and calcified parenchyma were only identified by evaluator 3, who marked 4 and 6 examinations, respectively. It is also worth noting that evaluator 3 recorded observations for all examination areas, whereas evaluators 1 and 2 did not record any observations in the cavities and calcified parenchyma areas. Finally, Figure 8 showcases examples of observations for 4 different patients with detailed marking of their findings.

**Table 5:** Results of the evaluation of the findings by the 3 experts considering the AP^a^ and lateral CXRs^b^ without additional clinical information. Each data point of the table represents the number of patients where the evaluators reported 1 or more times the presence of the finding. The last row includes all the patients with any of the previous abnormalities. The order of the data comes from the assessment of the findings by evaluators 1/2/3 (N=218).

Results	Overall, n	Confirmed, n	Unconfirmed TB^c^, n	Unlikely TB, n
Number of patients	218	10	95	113
Airway compression or tracheal displacement or both	4/0/7	2/0/0	2/0/4	0/0/3
Lymphadenopathy	7/7/34	3/1/4	4/4/22	0/2/8
Hyperinflation	4/0/3	2/0/0	2/0/2	0/0/1
Air space opacification	31/42/52	7/7/7	22/30/33	2/5/12
Collapsed lung	5/1/9	0/0/2	4/1/6	1/0/1
Nodular picture	1/2/3	0/0/1	1/2/2	0/0/0
Interstitial opacification	7/1/28	1/0/2	5/1/16	1/0/10
Pleural effusion	5/6/4	0/0/0	5/6/4	0/0/0
Cavities	0/0/4	0/0/0	0/0/3	0/0/1
Calcified parenchyma	0/0/6	0/0/0	0/0/4	0/0/2
Any abnormality	41/46/92	8/7/9	30/33/54	3/6/29

^a^AP: anteroposterior.

^b^CXR: chest x-ray.

^c^TB: tuberculosis.

To gain a deeper understanding of how various evaluations influence the final diagnosis of TB, we examined the association between the assessments made by each evaluator, including the final evaluation and the initial diagnostic classification. The results of the chi-square test (see Table S2 in Multimedia Appendix 1) indicated that the most significant association for the CXR features was observed with the identification of air space opacification, yielding *χ*^2^_1_>20.38 and *P*<.001 for all evaluators. The second most noteworthy finding was the significant association of lymphadenopathies with the initial classification for evaluator 1 (*χ*^2^_1_=5.79, *P*=.02) and evaluator 3 (*χ*^2^_1_=11.88, *P*<.001). Additionally, the final evaluation showed a significant association with the initial classification, with *P* values of .02, .005, and <.001 for evaluators 1, 2, and 3. These findings are consistent with those presented in Table 5, which highlighted that these fields had the highest number of observations among the rest.

Finally, we investigated the agreement between evaluators using the Cohen kappa score for the interreader agreement for image quality, the global evaluation, and all the different findings (see Table S3 in Multimedia Appendix 1). Concerning image quality, we observed substantial agreement between evaluators 1 and 2 (κ=0.65), but only fair agreement between evaluators 1 and 3 (κ=0.33) and 2 and 3 (κ=0.31), primarily due to evaluator 3 assessing many more images as “poor quality.” The agreement for the global evaluation was very similar, with fair agreement ranging from 0.26 to 0.32. However, for the findings, we found that air space opacification exhibited a moderate to substantial Cohen kappa index, ranging from 0.54 to 0.67. The number of observations identified by the evaluators (as shown in Table 5) and the association with the initial classification (as demonstrated in Table S2 in Multimedia Appendix 1) underscored the significance of air space opacification as a crucial finding. Its large number of observations, strong association, and consistency between different evaluators emphasize its importance in the diagnosis process. Another field demonstrating moderate to substantial agreement was pleural effusion, with Cohen kappa scores ranging from 0.43 to 0.72. However, despite this strong agreement, there were fewer observations and a weaker association with the initial classification. Lymphadenopathies also emerged as an important finding in terms of observation and association, but the agreement was only slight, ranging between 0.13 and 0.21.

## Discussion

### Principal Findings

Store-and-forward telemedicine has emerged as a valuable solution for improving access to specialist and primary health care advice, leveraging technological advancements to overcome barriers in low-resource settings [13,14]. Our work showcases the potential application of this approach in assessing TB in young children in underserved areas, where the shortage of specialists and the challenges associated with TB assessment in this population may have a greater impact. The positive assessment of the telemedicine system, coupled with the reduced time needed for evaluation, further bolsters the case for utilizing telemedicine in diagnosing pulmonary TB. This not only ensures timely intervention but also promotes efficient health care delivery.

The low sensitivity of x-rays in identifying positive cases in our pilot study corroborates the challenges reported in diagnosing TB in children, as documented in other studies [23-27]. Limited research has offered detailed insights into the global sensitivity and specificity of CXR in young children for TB diagnosis. Kaguthi et al [24] reported sensitivities ranging from 50% to 75% and specificities between 72.9% and 85.2%. However, they acknowledged the imprecision in measuring sensitivity due to the limited number of definitive cases. Berteloot et al [27] reported higher sensitivities (71.4%) and lower specificities (50.0%), although the evaluation process involved a consensus and an older age group of children. Other studies [25,26] have also investigated the performance of TB diagnosis using CXR but focused on the most relevant findings to support the diagnosis [25,26]. Similar to those findings, in our results, lymphadenopathies, opacifications, and pleural effusions were the findings having the strongest association with positive evaluation (as indicated in Table S2 in Multimedia Appendix 1). Integrating a treatment-decision algorithm that incorporates clinical evidence, CXR findings, and the Xpert MTB/RIF assay (or its current version, Xpert MTB/RIF Ultra), as proposed by several studies [2,9], could enhance the performance of the diagnostic process and streamline treatment decisions. This approach could be considered in future developments.

### Comparison With Other Studies

In terms of interreader agreement, our findings align, to some extent, with other studies that have also reported slight to moderate agreement [24,25,28]. Kaguthi et al [24] reported poor agreement on abnormalities consistent with TB (κ=0.14) and moderate agreement (κ=0.26) on lymphadenopathy. However, their lower agreement results compared with ours could be attributed to the variability in expertise among the readers. Our results are more closely aligned with other studies in terms of the reader profile [25,27,28]. For instance, Palmer et al [25] reported a moderate agreement (κ>0.4) on specific features such as alveolar opacification, pleural effusion, expansile pneumonia, and enlarged perihilar lymph nodes. Similarly, Berteloot et al [27] reported a κ value of 0.36 between a radiologist and a pediatric pulmonologist. Lastly, Andronikou et al [28] presented a κ value of 0.5 among trained pediatric radiologists, although their data set included older children with a mean age of 9 years.

### Limitations

Our pilot study has several limitations. First, the number of confirmed cases is small, and the presence of some important features relevant to diagnosis by CXR, such as airway compression or tracheal displacement, nodular pattern, cavities, or calcified parenchyma, is also limited. This may explain the lack of a stronger association with the TB classification highlighted in other studies [23]. The evaluators’ performance was compared with the case definition, which includes abnormal CXR as one of the criteria for unconfirmed TB. As observed in analogous studies [27,28], our research was constrained by the limited number of studies and readers. Broader validation, including a wider range of studies and readers, may provide more robust insights into the agreement and performance of the evaluations. The expertise of our readers may not fully reflect the typical skill set available in resource-limited settings; however, this challenge can be overcome through the implementation of consensus classifications. Moreover, the approach of conducting double assessments by both nonexperts and experts has been successfully tested in other projects [29,30], suggesting its potential effectiveness in enhancing diagnostic accuracy. By incorporating these methods into our telemedicine platform, we can overcome limitations related to reader expertise and enhance the overall diagnostic process for pediatric TB in resource-limited settings.

Besides the current utilization of the platform as a diagnostic tool for remote evaluation of CXR examinations, we have planned its future use for the systematic assessment of data sets in clinical studies and as a labeling tool for TB findings present in CXR. This will facilitate the training of artificial intelligence segmentation and classification models. The inclusion of new data sets from multiple settings and the expansion of the number of readers will enable a comprehensive validation of the platform. Additionally, it is essential to view CXR as part of a broader diagnostic algorithm for pediatric TB, which includes assessing symptoms; signs of TB; exposure to a TB source patient; results from tests for *M. tuberculosis* infection (eg, tuberculin skin tests or interferon-gamma release assays), microbiological tests (eg, Xpert MTB/RIF, microscopy, or culture for *M. tuberculosis*), and any other relevant supporting tests [15]. Following this approach, we are considering leveraging the platform with a treatment-decision algorithm that incorporates clinical evidence and artificial intelligence models to enable automatic CXR scoring. This integration has the potential to significantly enhance the accuracy and efficiency of TB diagnosis in young children.

### Conclusions

TB remains a significant global health challenge, particularly among children, and the COVID-19 pandemic has intensified the situation. CXR imaging is crucial for diagnosis, severity assessment, and treatment response evaluation. In this study, we introduced a novel telemedicine web platform, BITScreen PTB, which utilizes CXR images and clinical information. Its purpose is to streamline remote reading and standardize pediatric TB examinations in resource-limited settings.

Our platform received positive feedback from users, and while there may be room for further improvements to address concerns about reliability and interface quality, it shows promise for future use. Our study underscores the potential of telemedicine platforms such as BITScreen PTB to enhance access to TB diagnosis in children, especially in low-resource settings. Additionally, the platform has the potential to serve as a labeling tool for CXRs to develop and integrate artificial intelligence models, which could enhance the accuracy and speed of TB diagnosis in children, particularly in resource-limited settings.

## Appendix

Multimedia Appendix 1 Global evaluation and assessment results with interrater agreement.

## Abbreviations

AP: anteroposterior
BCG: Bacillus Calmette-Guérin
BITScreen PTB: Biomedical Image Technologies Screen for Pediatric Tuberculosis
CISM: Manhiça Health Research Center
CXR: chest x-ray
LAT: lateral
MVC: Model-View-Controller
PA: posteroanterior
PPV: positive predictive value
SATVI: South African Tuberculosis Vaccine Initiative
TB: tuberculosis
TUQ: Telehealth Usability Questionnaire
WHO: World Health Organization
