# Supplementary material for: Chest X-Ray–Based Telemedicine Platform for Pediatric Tuberculosis Diagnosis in Low-Resource Settings: Development and Validation Study
Source: JMIR Pediatr Parent. 2024 Jul 1;7:e51743. doi: 10.2196/51743 (PMC11250038; doi:10.2196/51743)
Supplement: Multimedia Appendix 1 [file pediatrics_v7i1e51743_app1.docx]

**Multimedia Appendix 1:** Supplementary material

**Chest X-Ray Based Telemedicine Platform for Pediatric Tuberculosis Diagnosis in Low-Resource Settings: Development and Validation Study**

**Figure S1**. Result of the assessment of the image quality of the CXRs. On the left is the quality of the AP View and on the right the quality of the Lateral View. NA, NR stands for Not Acceptable, Not Readable.


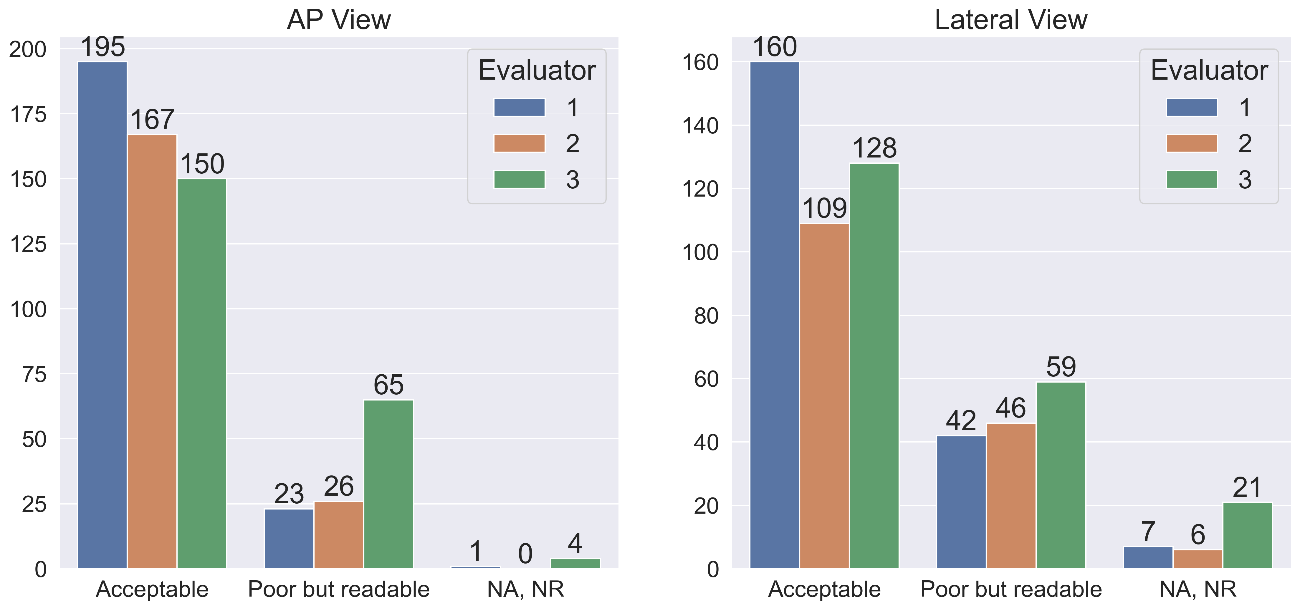


**Figure S2.** Examples of evaluations of image quality. On the left an AP view that was evaluated as “acceptable” by the three evaluators. On the right the Lateral view of an image evaluated as “not acceptable, not readable” by the three evaluators.


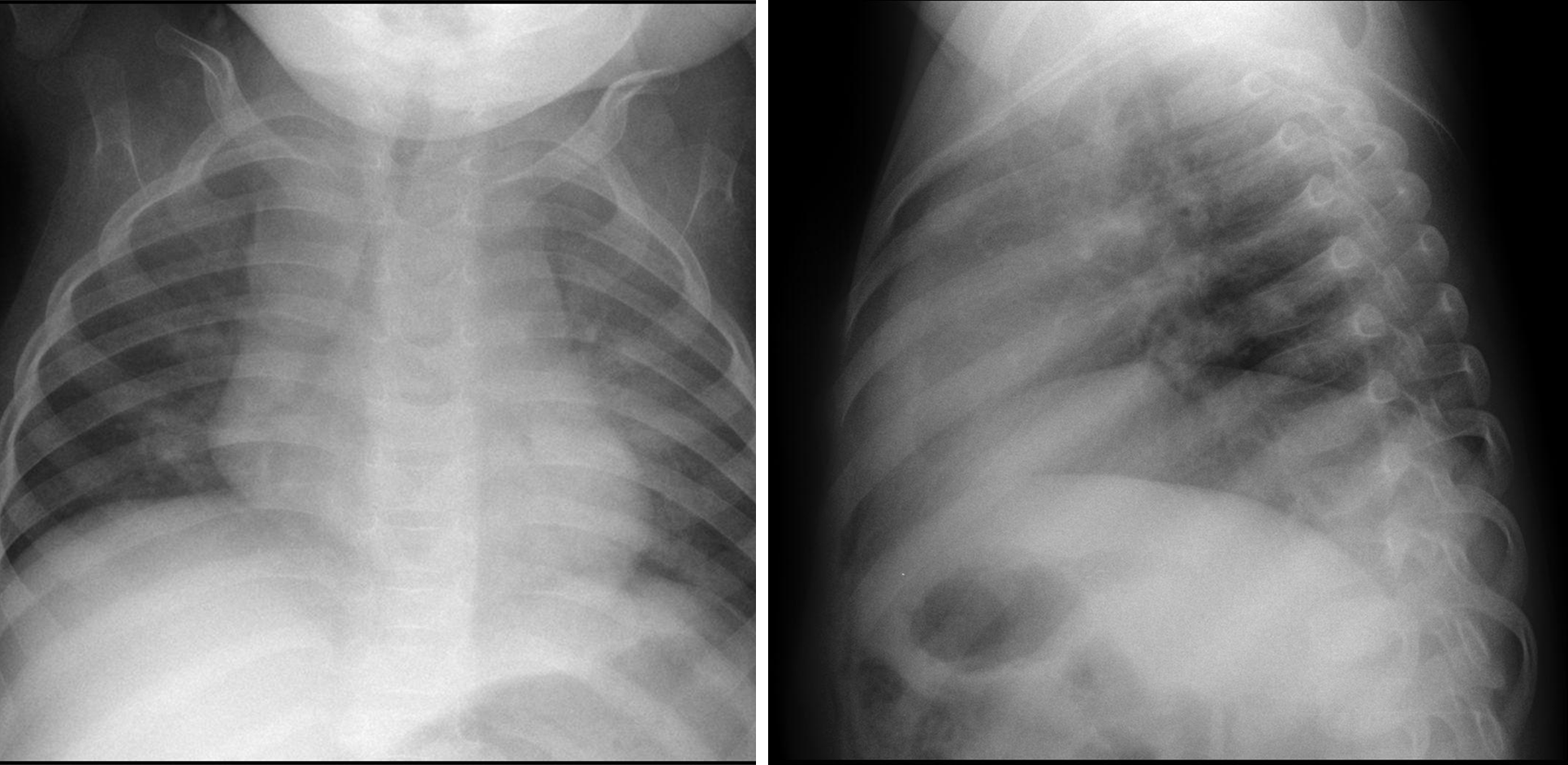


**Table S1.** Results of the global evaluation of the three evaluators (N=218)

|  | **Evaluator 1** | **Evaluator 2** | **Evaluator 3** |
| --- | --- | --- | --- |
|  |  |  |  |
| **Confirmed (n=10)** |  |  |  |
| Suggestive TB, n(%) | 3 (30) | 2 (20) | 4 (40) |
| Non-Suggestive TB, n(%) | 7(70) | 8 (80) | 5 (50) |
| Not evaluable, n(%) | 0 (0) | 0 (0) | 1 (10) |
| **Unconfirmed TB (n=95)** |  |  |  |
| Suggestive TB, n(%) | 14 (15) | 11 (20) | 25 (26) |
| Non-Suggestive TB, n(%) | 80 (84) | 84 (88) | 69 (73) |
| Not evaluable, n(%) | 1 (1) | 0 (0) | 1 (1) |
| **TB Unlikely (n=113)** |  |  |  |
| Suggestive TB, n(%) | 6 (5.3) | 2 (1.8) | 10 (8.8) |
| Non-Suggestive TB, n(%) | 106 (93.8) | 111 (98.2) | 102 (90.3) |
| Not evaluable, n(%) | 1 (0.9) | 0 (0) | 1 (0.9) |

**Table S2**. Chi-square test (Χ^2^_1_) and P values between the findings, the global evaluation, and the TB reference class of the three evaluations. We considered “Confirmed” and “Unconfirmed TB” as one class and “TB Unlikely” as other class.

|  | **Evaluator 1** | | **Evaluator 2** | | **Evaluator 3** | |
| --- | --- | --- | --- | --- | --- | --- |
|  | **Χ^2^_1_** | **P value** | **Χ^2^_1_** | **P value** | **Χ^2^_1_** | **P vaue** |
|  |  |  |  |  |  |  |
| **Airway compression and/or tracheal displacement** | 2.53 | .112 | - | - | 0.01 | .91 |
| **Lymphadenopathy** | 5.79 | .02 | 0.75 | 0.39 | 11.88 | <.001 |
| **Hyperinflation** | 2.53 | 0.11 | - | - | 0.01 | .94 |
| **Air space opacification** | 27.8 | <.001 | 31.27 | <.001 | 20.38 | <.001 |
| **Collapsed lung** | 0.98 | .32 | 0 | .97 | 4.72 | .03 |
| **Nodular picture** | 0 | .97 | 0.58 | .45 | 1.53 | .22 |
| **Interstitial opacification** | 2.68 | .10 | 0 | .97 | 2.75 | .10 |
| **Pleural effusion** | 3.59 | .06 | 4.68 | .03 | 2.56 | .11 |
| **Cavities** | - | - | - | - | 0.35 | .56 |
| **Global evaluation** | 5.74 | 0.02 | 7.98 | .005 | 12.09 | <.001 |

**Table S3.** Cohen Kappa (K) for the interobserver agreement of the global assessment and all the findings between Evaluator 1 and 2 (K 1-2), Evaluator 1 and 3 (K 1-3) and Evaluator 2 and 3 (K 2-3)

|  | **K 1-2** | **K 1-3** | **K 2-3** |
| --- | --- | --- | --- |
|  |  |  |  |
| **Airway compression and/or tracheal displacement** | 0 | 0 | 0 |
| **Lymphadenopathy** | 0.13 | 0.21 | 0.20 |
| **Hyperinflation** | 0 | 0.27 | 0 |
| **Air space opacification** | 0.67 | 0.54 | 0.63 |
| **Collapsed lung** | 0 | 0.26 | 0.19 |
| **Nodular picture** | 0 | 0.5 | 0 |
| **Interstitial opacification** | 0 | 0 | 0 |
| **Pleural effusion** | 0.72 | 0.43 | 0.59 |
| **Global evaluation** | 0.32 | 0.26 | 0.30 |
